# Targeting CX3CR1 Signaling Dynamics: A Critical Determinant in the Temporal Regulation of Post-Stroke Neurorepair

**DOI:** 10.3390/brainsci15070759

**Published:** 2025-07-17

**Authors:** Quan He, Tong Zhou, Quanwei He

**Affiliations:** 1 Tongji Hospital, Tongji Medical College, Huazhong University of Science and Technology, Wuhan 430030, China; u202113658@hust.edu.cn; 2 Department of Neurology, Union Hospital, Tongji Medical College, Huazhong University of Science and Technology, Wuhan 430022, China; m202476208@hust.edu.cn

**Keywords:** CX3CR1, ischemic stroke, CX3CL1/CX3CR1 axis, CX3CL1, microglia, neurorepair, neuroinflammation, biomarker

## Abstract

Ischemic stroke ranks among the top global causes of disability and mortality, with a highly dynamic pathological process. Post-stroke neuroinflammation, mediated by microglia, demonstrates a dual role in both injury and repair. The CX3CR1/CX3CL1 signaling axis, highly expressed in microglia, acts as a key regulator. This review examines the spatiotemporal dynamics of the axis across the stroke process and its involvement in neural repair. Crucially, this signaling pathway demonstrates stage-dependent functional duality: its cellular sources, receptor expression profiles, and functional consequences undergo temporally orchestrated shifts, manifesting coexisting or interconverting protective and damaging properties. Ignoring this dynamism compromises the therapeutic efficacy of targeted interventions. Thus, we propose a triple precision strategy of “stroke phase—biomarker—targeted intervention”. It uses specific biomarkers for precise staging and designs interventions based on each phase’s signaling characteristics. Despite challenges like biomarker validation, mechanistic exploration, and cross-species differences, integrating cutting-edge technologies such as spatial metabolomics and AI-driven dynamic modeling promises to shift stroke therapy toward personalized spatiotemporal programming. Temporally targeting CX3CR1 signaling may offer a key basis for developing next-generation precision neural repair strategies for stroke.

## 1. Introduction

Ischemic stroke represents a predominant global cause of mortality and long-term disability [1], with its core pathophysiology marked by the ischemic cascade—a dynamic sequence of excitotoxicity, oxidative stress, and neuroinflammation following cerebral hypoperfusion [2,3]. Microglia-mediated neuroinflammation exerts dual effects on post-stroke injury and repair [4]. CX3CR1, a fractalkine receptor highly expressed on microglia, plays a key role in modulating microglial activation, migration, and synaptic interactions [5]. By regulating microglial polarization and neuron–glia communication, CX3CR1 signaling significantly impacts neuroinflammation, neuronal survival, and regeneration [6], making it a promising therapeutic target. However, current research often uses static or single-timepoint analyses, failing to capture the pathway’s temporal dynamics [7]. The CX3CR1/CX3CL1 axis exhibits phase-dependent functional duality, with its cellular sources, signaling intensity, and biological consequences evolving across stroke progression, exhibiting both neuroprotective and neurotoxic manifestations at different stages [8]. Interventions ignoring this spatiotemporal dimension risk suboptimal outcomes. This knowledge gap in spatiotemporal dynamics impedes therapeutic advancement.

This narrative review rigorously adheres to the SANRA (Scale for the Assessment of Narrative Review Articles) guidelines. Through a comprehensive literature search in PubMed databases through June 2025, we systematically elucidate the spatiotemporal regulatory mechanisms of the CX3CR1/CX3CL1 signaling axis across the full continuum of ischemic stroke and its central role in neural repair processes. This review analyzes the temporal reprogramming of this signaling pathway across critical pathological phases and proposes a phase-stratified therapeutic framework. We explore innovative approaches using biomarker-guided staging to implement targeted interventions while addressing current research challenges and future translational opportunities. By integrating advances across neuroimmunology and systems biology, this work establishes a temporally sequenced targeting strategy for CX3CR1 modulation, providing a foundational framework for next-generation precision stroke therapeutics.

## 2. Spatiotemporal Dynamics and Dual Regulatory Roles of the CX3CR1/CX3CL1 Signaling Axis in Post-Stroke Pathology

The pathological progression of ischemic stroke exhibits highly dynamic temporal evolution. Although standardized time boundaries remain undefined in the current literature, it is conventionally categorized as follows [9,10,11,12]: 1. Hyperacute phase: <24 h post-onset, with critical therapeutic windows at ≤4.5 and ≤6 h [13]. 2. Acute phase: 24 h to 7 days [14,15] (some definitions emphasize the early subphase: days 1–3 [16]). 3. Subacute phase: 1 week to 3 months, subdivided into early (1–2 weeks) and late (2 weeks–3 months) stages [17,18]. 4. Chronic phase: >3 months [15,16]. Within this cascade, the CX3CR1/CX3CL1 signaling axis serves as a core molecular driver of pathological phase transitions due to its spatiotemporal-specific expression and functional variations [8,19]. The precise dissection of CX3CR1/CX3CL1 spatiotemporal dynamics requires advanced technologies: single-cell RNA sequencing (scRNA-seq) reveals molecular heterogeneity and temporal evolution of CX3CR1-expressing myeloid subsets post-stroke [20,21,22,23], while spatial transcriptomics captures astrocyte-derived CX3CL1 enrichment gradients within critical repair regions [24,25]. These spatial patterns drive localized activation of CX3CR1^+^ myeloid cells toward a reparative phenotype [26,27]. Although systematic quantitative data remain limited, convergent qualitative findings from multiple studies deploying these technologies establish the molecular and cellular foundations for elucidating how the CX3CL1/CX3CR1 axis drives its stage-dependent dual functions through spatiotemporally precise regulation.

### 2.1. Hyperacute Phase: Injury Perception and Emergency Response

Within hours of stroke onset, neurons in the ischemic core undergo rapid disintegration, releasing abundant soluble CX3CL1. This triggers a steep local CX3CL1 concentration gradient that functions as a critical “Find-Me” chemotactic signal to recruit phagocytic cells [6,7,28,29]. Concurrently, resident microglia in the peri-infarct zone undergo rapid but transient upregulation of CX3CR1 expression—a spatiotemporally constrained response essential for damage surveillance [5,8].

This spatiotemporal coordination—where CX3CL1 release in the ischemic core coincides with CX3CR1 upregulation in peri-infarct zones—enables the CX3CR1 pathway to mediate injury surveillance [30,31,32]. Activation of the signaling axis induces the transition of peri-infarct microglia from quiescence to activation. Microglial migratory capacity is significantly enhanced through CX3CR1-AKT signaling [33,34], guiding rapid cellular accumulation toward the injury core. At the lesion site, activated microglia initiate essential neuroprotective functions: cellular debris is phagocytosed [5,8], and hazardous substances are cleared. This process represents the beneficial onset of neuroinflammation, serving to contain damage progression.

However, excessive or sustained activation of this signaling axis at the hyperacute stage underlies its dangerous functional duality [8,35]. Current evidence suggests that functional transition of this axis may be driven by multifactorial mechanisms. We postulate that high concentrations of soluble CX3CL1 could potentially enhance receptor-binding efficiency through signaling properties distinct from membrane-bound ligands, potentially inducing receptor hyperactivation [36]. Concurrently, alterations in receptor conformational states—such as subtle helix VI shifts post-CX3CL1 binding—might modulate signal transduction intensity [37]. Crucially, divergent activation patterns of downstream effectors appear to underlie functional dichotomies: CX3CR1-mediated AKT phosphorylation likely promotes neuroprotective migration during initial signaling [38], while under chronic overactivation, identical phospho-effectors may excessively amplify migratory capacity and potentially trigger pro-inflammatory cascades [39]. This inflammatory shift could recruit monocytes and release mediators [40], presumably elevating local oxidative stress. Collectively, these mechanisms might drive microenvironmental evolution from transient debris-clearing repair toward acute neurotoxic inflammation [41,42], potentially exacerbating secondary brain injury.

### 2.2. Acute Phase: Dual-Phase Regulation of Neuroinflammation

As stroke pathology progresses into the acute phase (24 h–7 d), the CX3CR1/CX3CL1 axis undergoes critical spatiotemporal reprogramming [43,44,45,46]. Its regulatory function manifests dual-phase characteristics: acting as both an amplifier of inflammatory cascades and an intrinsic brake mechanism on neurotoxic overactivation [47,48,49,50,51].

The ischemic core and penumbral microvascular beds constitute the spatial epicenter of acute-phase pathology. Within these regions, hypoperfusion-induced low shear stress synergizes with early inflammatory mediators (e.g., TNF-α) to trigger robust CX3CL1 upregulation on vascular endothelia [43,44,45]. This endothelial-derived chemokine serves as a potent recruitment signal for CX3CR1-expressing peripheral immune cells—particularly pro-inflammatory Ly6C^+^ monocytes—directing their infiltration into ischemic parenchyma [46].

Thus, CX3CR1 signaling acts as a direct amplifier of inflammatory storms: Endothelially upregulated CX3CL1 specifically drives peripheral CX3CR1^+^Ly6C^+^ pro-inflammatory monocytes to initiate the classical adhesion cascade—including rolling, firm adhesion, and transendothelial migration—efficiently mediating their infiltration into ischemic brain parenchyma [52,53]. These newly recruited Ly6C^+^ monocytes rapidly expand within the ischemic core and penumbra, differentiating into pro-inflammatory macrophages (monocyte-derived macrophages, MDMs) [54]. Upon cerebral entry, this macrophage-differentiated population becomes the primary instigator of neuroinflammatory storms, dominantly releasing potent pro-inflammatory cytokines such as IL-1β and TNF-α [47,48]. These newly differentiated macrophages significantly amplify neuroinflammatory cascades, exacerbating neuronal injury, blood–brain barrier disruption, and secondary brain tissue damage.

Simultaneously, the CX3CR1 signaling pathway in resident microglia exhibits a unique “braking function”: It specifically activates microglia, driving their polarization toward an M2-like phenotype characterized by anti-inflammatory and neuroprotective properties. This CX3CR1-dependent rapid phenotypic reprogramming induces microglial secretion of key anti-inflammatory factors (e.g., IL-10), significantly suppressing local inflammatory storms and thereby modulating neuroinflammatory progression [49,50,51]. This reprogrammed state is recognized as the initiation point for inflammation self-limitation, actively suppressing inflammatory intensity while promoting tissue repair, thereby constraining early-stage neuroinflammatory exacerbation.

The coexistence of these seemingly opposing effects—pro-inflammatory amplification versus anti-inflammatory braking—epitomizes the bimodal regulatory nature of CX3CR1 signaling during the acute phase. The homeostatic equilibrium of this signaling axis critically depends on spatiotemporal cellular contexts and crosstalk with key pathways—particularly the complex co-regulatory interplay between CX3CR1 and TREM2 (triggering receptor expressed on myeloid cells 2). This synergy manifests through: 1. Co-expression modulation (e.g., synchronized downregulation in metabolic/drug models [55,56]). 2. Signaling pathway intersection (LRRK2-NFATc1 axis [57]). 3. Functional complementarity (TREM2-mediated phagocytosis promotion vs. CX3CR1-driven suppression of inflammatory hyperactivation [58,59]). These mechanisms co-regulate microglial phagocytic activity and jointly modulate disease-associated phenotypes—including DAM (disease-associated microglia) transition [60,61]—and the inflammation–phagocytosis equilibrium. The dynamic equilibrium is ultimately determinative for acute neuroinflammation magnitude/duration, directly influencing neuronal survival in the ischemic penumbra and overall tissue damage extent [62,63].

### 2.3. Subacute Phase: Neural Repair Activation

During the subacute phase (1 week to 3 months post-stroke), neuroinflammation gradually subsides while tissue repair mechanisms initiate. The CX3CR1/CX3CL1 axis undergoes functional repurposing: its spatiotemporal reprogramming primarily promotes neural repair [64,65,66], yet insufficient signaling intensity may constitute a novel therapeutic challenge [67,68,69,70].

As acute inflammation resolves, activated astrocytes—particularly in peri-infarct zones—supersede vascular endothelia as the dominant CX3CL1 source [5,71]. This astrocyte-dominated signaling marks a spatial shift from inflammatory to reparative programs. Within lesioned areas (ischemic core and periphery), microglia/macrophages exhibiting sustained CX3CR1 expression emerge as primary responders to astrocyte-derived CX3CL1, executing neural repair functions [72]. Here, microglia function as dominant regulators—leveraging their CNS-resident immunity to directly drive tissue repair through anti-inflammatory polarization, neuroprotection, and angiogenesis [73,74]. In parallel, macrophages—particularly MDMs—operate as auxiliary units that secrete anti-inflammatory mediators (e.g., IL-13) to dampen inflammation and bolster neuroregeneration, thereby reinforcing microglia-led reparative cascades [75,76].

During this phase, the CX3CR1/CX3CL1 axis centrally coordinates multifaceted repair processes: 1. Debris clearance: CX3CR1 signaling drives microglia/macrophages toward a pro-phagocytic phenotype that efficiently clears injury residues (e.g., myelin debris), establishing a permissive microenvironment for remodeling [6,66]. 2. Angiogenesis induction: CX3CR1-high macrophages secrete VEGF, directly stimulating vascular remodeling and perfusion recovery in peri-infarct zones [77]. 3. Neurogenic support: Neuron-/glial-derived CX3CL1 specifically activates neural progenitor cells (NPCs) via CX3CR1 [78,79,80], enhancing survival and promoting endogenous neurogenesis for circuit reconstruction [63,64,65,66,81]. At the molecular level, extracellular regulated protein kinases (ERK) pathway activation downstream of CX3CR1 constitutes the core mechanism mediating these repair functions [80]. ERK upregulates neurotrophic factor release [64,81], supporting neuronal survival, axonal regeneration, and synaptic plasticity.

However, dysfunction or disruption of the CX3CR1/CX3CL1 axis during this repair-oriented shift carries significant risks. Impaired signaling (e.g., CX3CR1 inhibition or insufficient CX3CL1) may trigger maladaptive astrocyte activation [67], culminating in dense gliotic scar formation [68]. These scars severely impede axonal regeneration, synaptic remodeling, and tissue reintegration [69,70], ultimately compromising long-term functional recovery potential.

### 2.4. Chronic Phase: Perpetuating Inflammation and Secondary Degeneration

During the chronic phase (>3 months post-stroke), persistent tissue remodeling occurs and neurological sequelae are manifested [63]. The CX3CR1/CX3CL1 axis exhibits spatiotemporally sustained high-level activation—prolonging subacute-phase neurorestorative effects during early stages [4], yet its chronic hyperactivation triggers a paradoxical functional shift: transitioning from pro-repair potential to a driver of persistent neurological deterioration [82].

During this phase, surviving neurons in lesioned cores and adjacent regions, alongside activated astrocytes, persistently produce CX3CL1 [24,83,84] with diffuse spatial distribution. Microglial CX3CR1 expression is stably maintained at elevated levels [85,86], establishing these cells as the primary chronic responders to sustained CX3CL1 signaling.

Sustained CX3CR1 hyperactivity at this stage transitions from reparative to pathogenic, becoming a core driver of chronic neuroinflammation, circuit damage, and secondary neurodegeneration. Its multifaceted destructive mechanisms include the following: 1. Pathological synaptic pruning: Elevated CX3CR1 signaling abnormally activates microglial phagocytosis and complement cascades [55,87], inducing excessive elimination of synaptic components through uncontrolled pruning. This results in widespread synaptic loss [88,89,90], disrupting neural circuit integrity. 2. Chronic pro-inflammatory microenvironment: CX3CR1 drives persistent microglial pro-inflammatory polarization [51,91,92], establishing self-perpetuating neurotoxic microenvironments that amplify neural damage. 3. Accelerated proteinopathy: CX3CR1 hyperactivity accelerates pathological protein aggregation (e.g., amyloid β protein [Aβ] deposition) [93]. Conversely, CX3CR1 deficiency delays Aβ pathology in experimental models [94], indicating its role in promoting post-stroke Alzheimer’s-like neurodegeneration.

Chronic-phase CX3CR1 signaling dysfunction correlates directly with long-term neurocognitive outcomes in stroke survivors. Human genetic studies confirm that the CX3CR1 T280M loss-of-function polymorphism significantly elevates risk for post-stroke cognitive impairment and dementia [63,95,96,97]. This evidence achieves the following: 1. Validates CX3CR1’s central role in maintaining neural circuit integrity and resisting neurodegeneration. 2. Highlights the necessity for precise chronic-phase regulation to prevent detrimental neurocognitive outcomes. These clinical data establish CX3CR1 signaling as a critical molecular determinant of long-term neurological prognosis after stroke.

The spatiotemporal reprogramming and functional duality of the CX3CR1/CX3CL1 signaling axis across stroke progression are systematically characterized in Table 1:

It is now well established that the CX3CR1 signaling axis plays a stage-dependent dual role following stroke [8,36]. However, current research predominantly addresses phenomenological observations, leaving critical mechanistic knowledge gaps: 1. Ligand concentration threshold: How does soluble CX3CL1 (sCX3CL1) switch functional states via concentration gradients during the hyperacute phase? 2. Receptor conformational reprogramming: Do post-stroke CX3CR1 post-translational modifications (e.g., phosphorylation/glycosylation) influence signaling bias? 3. Co-receptor interaction networks: How does spatiotemporal co-localization with receptors like TREM2 and CCR2 modulate CX3CR1 signal output? 4. Downstream signal sorting: Why does the same receptor activate pro-inflammatory versus neuroreparative pathways across distinct pathological stages? Addressing these mechanistic voids represents an urgent research priority. Notably, partial mechanisms have been elucidated. The CX3CL1/CX3CR1 axis confers neuroprotection by inhibiting NLRP3 (NOD-like receptor thermal protein domain associated protein 3) inflammasome activation [6] through two complementary pathways: 1. PPM1A (magnesium-dependent phosphatase 1 A) phosphatase-mediated suppression: CX3CR1 signaling activates PPM1A, which binds NLRP3 to disrupt its complex formation with ASC (apoptosis-associated speck-like protein containing a CARD) and pro-caspase-1, directly blocking inflammasome assembly [98]. 2. NF-κB (nuclear factor kappa-B) pathway inhibition: CX3CR1 activation suppresses NF-κB signaling, reducing pro-inflammatory cytokine release and indirectly impeding NLRP3 oligomerization [99]. Conversely, NLRP3 inflammasome activation may exacerbate neuroinflammation, counteracting CX3CR1-mediated neuroprotection [6]. This reveals a bidirectional regulatory circuit—the PPM1A/NF-κB/CX3CR1-NLRP3 axis—that orchestrates stage-specific transitions between pro-inflammatory and anti-inflammatory/repair responses post-stroke. Future studies must comprehensively dissect CX3CR1’s core regulatory mechanisms to fully decode its stage-dependent duality.

## 3. Phase-Adapted Therapeutic Strategies Targeting CX3CR1

Given the stage-dependent functional duality of CX3CR1 signaling and its dynamic evolution across stroke pathology, therapeutic interventions must adhere to strict temporal precision. This section synthesizes advances in time-window-specific targeting strategies, examining key technological innovations and clinical translation pathways for phase-adapted modulation of the CX3CR1 axis.

### 3.1. Timeliness Challenges of Existing Intervention Strategies

Current evidence indicates that systemic CX3CR1 antagonism during the hyperacute phase compromises microglial neuroprotection by inhibiting migration and polarization toward acute surveillance phenotypes [100,101,102]. Critically, such intervention exacerbates early blood–brain barrier (BBB) disruption [103,104,105]. These findings collectively demonstrate that CX3CR1-targeted interventions during this phase may not only impair microglia-mediated neuroprotective mechanisms but also accelerate neural injury cascades and pathological progression.

During the acute phase, CX3CR1 antagonism significantly suppresses microglial phagocytic function [51,106], resulting in the pathological accumulation of necrotic neuronal debris within injured areas [107,108]. This debris accumulation amplifies neuroinflammatory cascades and exacerbates secondary tissue damage [38,51]. Consequently, CX3CR1 inhibition during this phase not only fails to modulate inflammation effectively but also intensifies secondary neural injury by impairing dead cell clearance.

The administration of exogenous CX3CL1 agonists (e.g., recombinant CX3CL1 [rCX3CL1] protein or fractalkine [FKN]-Fc fusion protein) during the subacute phase effectively induces microglial polarization toward a reparative phenotype [29,109]. This strategy enhances microglial clearance efficiency for hematomas and cellular debris while promoting vascular remodeling and improving functional recovery [6,110]. These findings establish the subacute phase as a critical therapeutic window for CX3CR1 pathway activation to maximize microglia-mediated neuroprotection.

While sustained, long-term CX3CR1 inhibition during the chronic phase reduces neuroinflammatory responses [111], concurrent suppression of essential synaptic remodeling and neural regeneration processes occurs [82,112]. Murine model studies confirm that cognitive recovery is significantly delayed by prolonged CX3CR1 pathway antagonism [46]. Thus, chronic, long-term monotherapy targeting CX3CR1 may alleviate inflammation while inducing inhibition of neural plasticity, ultimately resulting in hindered functional recovery.

Collectively, this evidence confirms that CX3CR1-targeted stroke therapies must incorporate strict temporal specificity. Interventions at non-optimal timepoints—such as pathway inhibition during hyperacute/acute phases or simplistic chronic-phase suppression—risk inducing counterproductive outcomes. Conversely, pathway activation during the subacute phase maximizes reparative potential. These findings underscore the imperative for temporally programmed therapeutics.

### 3.2. Biomarkers Drive Precision Staging

In stroke management, precise pathological staging is fundamental for targeted CX3CR1/CX3CL1 modulation and essential for elucidating disease mechanisms, optimizing therapies, and predicting outcomes. Conventional staging—primarily based on symptom onset and imaging findings [113]—is limited by inadequate sensitivity [114] and temporal lag [115]. Emerging stage-specific biomarkers, which directly mirror molecular pathology, are now driving a paradigm shift toward precision staging characterized by enhanced accuracy, objectivity, and personalization.

During stroke’s hyperacute phase, blood–brain barrier (BBB) disruption constitutes the core pathological event [116,117]. Serum matrix metalloproteinase-9 (MMP-9) levels exhibit a sharp increase during this phase [118,119]. MMP-9 specifically degrades endothelial tight junction proteins (e.g., occludin and zonula occludens-1) and type IV collagen in the basement membrane while activating other protease systems [120,121,122], functioning as a key effector of BBB damage [118,121]. Concurrently, CX3CL1 mediates microvascular endothelial barrier dysfunction through the Src/P115-RhoGEF/ROCK pathway [123]. Therefore, dynamic changes in serum MMP-9 and CX3CL1 levels provide sensitive humoral markers for detecting hyperacute-phase BBB injury.

As previously stated, the CX3CR1 and TREM2 pathways exhibit precise synergistic regulation that jointly controls acute neuroinflammatory dynamics after stroke [124]. Stroke model studies demonstrate that TREM2 functional defects directly inactivate microglial phagocytic capacity [125,126]. This dysfunction consistently coincides with significant downregulation of the scavenger receptor CD36 [127,128], thereby impairing CD36-mediated phagocytic clearance—confirming TREM2’s essential role in sustaining this critical process. Critically, Kota Kurisu et al. revealed that TREM2 deficiency extends beyond microglia to impair phagocytic function in myeloid-derived immune cells, including post-stroke infiltrating macrophages [129], establishing TREM2’s central regulatory role in myeloid responses. Given TREM2’s pivotal regulation of microglial activity and neuroinflammatory cascades, its expression dynamics offer a critical window into neuroinflammatory states. Current cerebrospinal fluid (CSF) TREM2 measurements have generated substantial biomarker data [122,130], supporting its potential use as a bodily fluid biomarker for assessing post-stroke inflammatory responses and pathological burden.

Non-invasive imaging techniques now enable in vivo dynamic monitoring of microglial responses. Lucio D’Anna et al. utilized the PET tracer [^11^C]PBR28 targeting the 18 kDa translocator protein (TSPO)—highly expressed on microglia/macrophage mitochondria—demonstrating significantly elevated tracer uptake in subacute-phase ischemic stroke lesions [131]. This directly visualizes inflammatory cell aggregation. Complementarily, Alessandro Villa et al. synthesized a novel carbon-11-labeled P2RY12-targeted PET tracer [132], leveraging P2RY12 upregulation during anti-inflammatory responses. This tracer provides a potential tool for precisely assessing microglial subpopulations with reparative phenotypes in the subacute phase. Elevated P2RY12 expression serves as a key molecular signature of microglial “homeostatic” or “reparative” states, correlating with phagocytic clearance and neuroprotective functions [133,134]. Notably, CX3CR1 signaling acts as a core molecular switch driving microglial transition toward this reparative phenotype during this phase [135]. Activation of pathways such as PI3K-Akt likely constitutes a critical mechanism underlying its repair-promoting functions—including pro-angiogenic effects and neurotrophic factor release [136]. Collectively, these advances reinforce the consensus that finely regulated microglial responses are indispensable for neural tissue repair and remodeling during stroke’s subacute phase [137,138].

Neurofilament light chain (NfL) represents the gold standard biomarker for neuronal axonal injury. Its levels in CSF or serum exhibit significant elevation across neurological disorders including stroke, with established utility as a key indicator of axonal damage [139,140,141]. Notably, Magnus Gisslén et al. demonstrated that in neurological diseases (e.g., HIV-associated neurocognitive disorders), CSF soluble TREM2 (sTREM2) levels increase with disease severity while independently correlating with NfL levels reflecting neuronal injury [142]. This indicates that TREM2 signaling and microglial function critically modulate neuronal damage and repair mechanisms. Further evidence reveals that TREM2-mediated microglial activity participates in synaptic loss regulation during brain injury and disease [143], providing key insights into the molecular basis of post-stroke neurological deficits and recovery. Substantial evidence indicates that sustained CX3CR1 overactivation during the chronic phase constitutes a core pathological mechanism: It facilitates microglia-mediated pathological synaptic pruning (through phagocytosis of synaptic components) [144,145], maintains pro-inflammatory microenvironments [51,146], and may accelerate amyloid pathology [93], ultimately causing neuronal damage manifested by elevated NfL. Dynamic NfL monitoring combined with complementary biomarkers (e.g., sTREM2) will enable precision staging of neurological damage severity and prediction of long-term recovery potential.

In summary, biomarkers across stroke progression provide multidimensional molecular evidence for precise pathological staging: 1. Hyperacute phase: MMP-9 and CX3CL1 as early-warning markers of BBB disruption. 2. Acute phase: TREM2 reflecting pro-/anti-inflammatory dynamics. 3. Subacute phase: TSPO/PET imaging capturing microglial/macrophage activation/repair status, alongside emerging P2RY12-targeted PET tracers. 4. Chronic phase: NfL quantifying axonal damage and neural recovery. These biomarkers collectively unveil core stage-specific pathophysiological mechanisms while driving personalized therapeutic strategies, thereby establishing a foundation for biomarker-guided precision staging and targeted interventions in stroke management.

### 3.3. Cutting-Edge Technologies Enable Precise Interventions

Emerging technologies have now overcome the core spatiotemporal constraints of traditional stroke drug delivery by enabling lesion-specific targeting and chronotherapeutic delivery control, thus making precision interventions feasible.

pH-responsive nanoparticles enable targeted delivery of CX3CR1 modulators (agonists/antagonists) to the acidic microenvironment of ischemic brain regions [147,148,149,150,151]. This system triggers localized drug release under acidic pH conditions, significantly enhancing drug concentration at lesion sites while reducing systemic exposure and toxicity risks [152,153]. These findings demonstrate that mimicking pathological microenvironment characteristics achieves spatiotemporal-specific drug delivery, thereby overcoming limitations of traditional systemic administration in dynamically regulating CX3CR1 function.

Adeno-associated virus (AAV)-based gene regulation systems enable conditional knockout of the CX3CR1 gene in microglia within defined time windows (e.g., post-acute phase) through light-controlled or chemically inducible promoters [101,154,155]. This strategy effectively avoids impairing CX3CR1’s protective functions during hyperacute/acute phases while precisely inhibiting chronic-phase pathogenic signaling [63,86,156], achieving spatiotemporal gene expression control. Collectively, gene editing coupled with inducible systems creates a tunable molecular switch for dynamically regulating CX3CR1’s dual neuroprotective/neurotoxic effects.

Moreover, novel CX3CR1-targeted monoclonal antibodies [157] and aptamers [158]—characterized by small molecular weight, high stability, and low immunogenicity [159,160]—exhibit highly specific CX3CR1-modulating capabilities with promising translational potential. These molecules have demonstrated utility in cancer immunotherapy [157] and inflammatory regulation [158], positioning them as promising candidates for future stroke therapeutics targeting CX3CR1.

### 3.4. Clinical Translation: Triple Precision Strategy

Integrating stroke pathology staging, biomarker profiling, and targeted interventions, we propose a triple precision strategy for clinical translation:

#### 3.4.1. Hyperacute Phase: sCX3CL1/MMP-9 + Vascular Recanalization/Immune Regulation

Biomarkers-guided: Elevated serum levels of sCX3CL1 and MMP-9 serve as critical biomarkers for predicting blood–brain barrier disruption risk [118,121,123]. Dynamic monitoring of these biomarkers enables identification of the optimal therapeutic window for CX3CR1-targeted interventions, thereby preventing exacerbation of secondary neural damage while supporting vascular recanalization and immune homeostasis restoration.

Precision intervention strategies: The core therapeutic challenge in the hyperacute phase centers on balancing vascular recanalization with inflammation control, rather than direct CX3CR1 targeting. Current evidence consistently establishes vascular recanalization—including intravenous thrombolysis within 4.5 h or endovascular thrombectomy within 6 h [161,162]—as the primary intervention [163]. Complementarily, immunomodulatory therapies targeting specific pro-inflammatory mediators (e.g., TNF-α, IL-1β) are confirmed as essential adjunctive strategies during this phase [164,165,166]. Thus, the optimal approach prioritizes vascular recanalization as the cornerstone treatment, augmented by short-term, non-CX3CR1-targeted immunomodulation to suppress excessive early inflammatory cascades.

#### 3.4.2. Acute Phase: sTREM2/CD36 + rCX3CL1 Nanocarrier Delivery

Biomarkers-guided: Based on our earlier conclusions, significant reductions in CSF levels of sTREM2 and scavenger receptor CD36 serve as validated biomarkers for microglial phagocytic dysfunction [125,126,127,128]. The downregulation of sTREM2/CD36 not only reflects TREM2 pathway dysfunction but also indirectly suggests potential failure of CX3CR1 signaling to effectively drive microglial transition toward a high-efficiency phagocytic phenotype—or the predominance of its pro-inflammatory functions [167,168]. Consequently, monitoring sTREM2/CD36 dynamics offers a crucial window for assessing whether the CX3CR1 pathway resides in a repair-favorable “braking state” that promotes anti-inflammatory/pro-phagocytic phenotypes.

Precision intervention strategies: At precisely this crucial window of CX3CR1 reparative insufficiency, we propose exogenous augmentation of CX3CR1 signaling to directly counteract its dysfunction. This intervention drives microglia/macrophage polarization toward a pro-phagocytic, anti-inflammatory phenotype, thereby removing necrotic debris and curbing inflammatory cascade amplification. To achieve spatial precision, pH-responsive nanocarriers [169,170,171] enable targeted delivery of rCX3CL1. These systems not only efficiently traverse the compromised blood–brain barrier [172] but also trigger localized drug release within the acidic microenvironment of the ischemic penumbra [173,174]. This microenvironment-responsive mechanism maximizes local drug concentration, potentiates reparative signaling pathway activation, and minimizes systemic adverse effects.

#### 3.4.3. Subacute Phase: PET-MRI PBR28/P2RY12 + FKN-Fc/MSCs

Biomarkers-guided: When PET-MRI dual-modality imaging reveals significantly elevated [^11^C]PBR28 uptake in lesioned areas alongside increased microglial P2RY12 expression, this biomarker profile signifies reparative microglial activation [131,132] and marks the transition to the subacute disease phase [137,138]. Concurrently, the CX3CR1 pathway resides within a therapeutically actionable “time window” during which it can be effectively activated to promote neural repair [135]. Dynamic monitoring of these parameters enables precise identification of the endogenous repair initiation window, providing the basis for combined therapeutic strategies that maximize synergistic effects between endogenous mechanisms and exogenous interventions.

Precision intervention strategy: Mesenchymal stem cells (MSCs) demonstrate significant pro-angiogenic capacity [175,176], with confirmed therapeutic efficacy via intravenous administration during the stroke’s subacute phase [177,178]. Building on this evidence and prior conclusions, a combined intervention strategy can be implemented during this phase to maximize synergistic effects: intravenous co-administration of FKN-Fc fusion protein with MSCs [179]. This approach aims to exogenously enhance CX3CR1 signaling, synergizing with endogenous repair signals to consolidate and amplify microglial reparative phenotypes. Concurrently, leveraging MSC paracrine actions, it coordinately promotes angiogenesis, neurogenesis, and tissue remodeling [180,181,182], ultimately accelerating functional recovery in the subacute phase.

#### 3.4.4. Chronic Phase: sTREM2/NfL + AAV Delivery of CRISPR-Cas9

Biomarkers-guided: CSF levels of sTREM2, as a cleavage product of TREM2, serve as a potential biomarker for microglia-mediated synaptic damage induced by signaling pathways such as neuroinflammation [142,143]. Concurrently, elevated NfL levels directly correlate with neuronal damage pathology [139,140,141]. Therefore, persistently elevated sTREM2 levels in CSF coupled with aberrant NfL elevation collectively signal ongoing synaptic injury [183] and chronic neuroinflammation [184]. This biomarker profile indicates that CX3CR1 signaling has transitioned from its early protective/reparative role into a chronic pathogenic driver [93,144,185]. Dynamic monitoring of CSF sTREM2 and NfL levels provides a critical temporal window reference for targeted chronic-phase therapy. Within this window, reversible gene regulation strategies can be applied using AAV vector technology to achieve precise reprogramming of microglial functional homeostasis.

Precision intervention strategies: Chronic-phase interventions must overcome prior limitations of purely inhibitory approaches. Upon detecting this detrimental biomarker shift (sTREM2↑ + NfL↑), precisely and reversibly inhibiting CX3CR1 signaling emerges as a theoretically viable strategy. This intervention aims to block its pathogenic drivers—pathological synaptic pruning, chronic neuroinflammation, and neurodegeneration—thereby preserving neural circuits and improving long-term prognosis. Critical research demonstrates that the HEXB gene’s natural/minimal promoter drives specific and stable expression in microglia under physiological and pathological conditions, making it ideal for CNS-targeted AAV therapy [186]. The team led by Minmin Luo pioneered the AAV-MG1.2 vector, which effectively delivers CRISPR-Cas9 for microglial gene knockout [187]. Concurrently, studies confirm the HEXB locus enables tamoxifen-induced Cre recombinase-dependent gene manipulation [188]. Building on these findings, we propose a chronic-phase strategy: use AAV-MG1.2 vectors carrying the HEXB promoter-driven CRISPR-Cas9 system to target CX3CR1. Tamoxifen induction then achieves spatiotemporal-specific, reversible CX3CR1 knockout—suppressing pathological overactivation while preserving basal physiological functions. While AAV-CRISPR targeting of CX3CR1 holds significant therapeutic promise, its broad expression in peripheral monocytes [189] and specific neuronal populations [190] necessitates vigilance against potential systemic off-target effects (e.g., immune dysregulation) [191,192] and perturbation of compensatory chemokine pathways (e.g., CCR2 upregulation) [193]. Consequently, optimizing delivery systems and dosage parameters represents a critical research priority.

The proposed clinical translation framework is systematically summarized in Table 2:

## 4. Challenges and Future Directions

### 4.1. Bottlenecks in Clinical Translation

Implementing disease-stage-based precision interventions faces a core challenge: translating candidate biomarkers from mechanistic research to clinical application. The feasibility of this review’s advocated “Triple Precision Strategy” critically depends on the real-time accuracy of biomarker monitoring and clearly established pathological-stage thresholds within the framework. However, the stroke field currently lacks authoritative standardized definitions and detection thresholds for key biomarkers, as validated through large-scale clinical cohorts. Moreover, stage-specific therapeutic challenges manifest with distinct priorities: Hyperacute phase interventions carry significant immunosuppression risks [194,195], where imprecise systemic cytokine modulation may compromise host defense mechanisms [196,197]; chronic phase management faces predominant delivery hurdles—illustrated by fibrotic scarring in infarct cores that severely impairs vector penetration [198,199] despite AAV-MG1.2 vector’s microglia-targeting capability; ultimately, gene-editing technologies employed in precision interventions inevitably raise complex ethical considerations. Although the “Triple Precision Strategy” theoretically enables accurate staging across stroke phases, its clinical implementation faces significant practical constraints—including reliance on specialized instrumentation, time-intensive procedures, and substantial costs. Consequently, translating this strategy requires critical balancing of diagnostic precision against operational efficiency and economic feasibility. Future research should develop cost-effective implementation protocols that maintain staging accuracy while enhancing real-world deployability.

Beyond the critical knowledge gaps in core CX3CR1 signaling mechanisms previously noted, significant uncertainties persist in upstream regulatory pathways governing this axis [8]. Four fundamental knowledge gaps require urgent resolution: 1. Cell-type-specific transcriptional control identifying core transcription factors and regulatory networks driving CX3CR1 expression across distinct cellular contexts (monocyte subsets, microglia, NK cells, neurons). 2. Transcriptional network plasticity elucidating how regulatory components dynamically adapt to cellular state transitions (resting → activated) and microenvironmental cues (inflammatory/metabolic signals). 3. Chemokine receptor crosstalk defining molecular mechanisms (heterodimerization, co-stimulation, or mutual inhibition) enabling functional integration with receptors like CCR2/CCR5. 4. Ligand-induced signaling diversification determining signal characteristics (intensity thresholds, duration) and decoding mechanisms underlying functional bias in CX3CL1/CX3CR1-mediated multifunctionality (chemotaxis, stable adhesion, cell survival/anti-apoptosis) [200,201]. These mechanistic gaps constitute critical barriers to therapeutic translation.

Meanwhile, current CX3CR1 research faces significant interspecies temporal scale differences in clinical translation [202]. CITE-seq (Cellular Indexing of Transcriptomes and Epitopes by Sequencing) analysis reveals distinct expression patterns of CX3CR1 across T-cell differentiation gradients in humans versus mice. In humans, CX3CR1 expression strongly correlates with CD8^+^ T-cell differentiation status and migratory/functional properties, serving as a key discriminator between CD8^+^ and CD4^+^ T-cell states. However, murine CX3CR1 primarily mediates microglial functions including neuroinflammation modulation and phagocytic activity [203]. Further investigation of human immune disorders uncovers the dynamic complexity of the CX3CL1/CX3CR1 axis: In systemic lupus erythematosus (SLE), CX3CR1 expression in B cells/plasmablasts is negatively regulated by Notch signaling [204]—a mechanism absent in murine models, indicating broader immune regulatory hierarchies in humans. Additionally, the CX3CR1-dependent neural repair window identified in rodent models [205] may not directly translate to human stroke patients due to longer disease courses with greater pathophysiological complexity. Cross-disease evidence—including systemic inflammatory disorders [206], low-grade gliomas (LGGs) [207], subarachnoid hemorrhage (SAH) [208], and aortic aneurysms (AAs) [40])—has elucidated the clinical translational potential of CX3CR1 through single-cell transcriptomics, genome-wide association studies (GWAS), or postmortem tissue analyses; however, direct clinical evidence defining optimal therapeutic time windows for CX3CR1-targeted interventions in ischemic stroke remains limited. To precisely define the human brain repair critical window, advanced models must be developed that authentically recapitulate human neuropathological features. Therefore, a research system integrating human brain organoids with high-resolution in vivo imaging technology [209] will provide a powerful tool for simulating the unique spatiotemporal dynamics of neural repair in the human brain.

The core scientific challenges surrounding CX3CR1 constitute critical bottlenecks impeding the clinical translation of stroke precision therapeutics. Overcoming these barriers demands a multidimensional integrated strategy: 1. Conduct rigorous large-scale clinical studies to validate and clinically implement key biomarkers. 2. Integrate dynamic CD4^+^/CD8^+^ ratio monitoring [210] into clinical protocols to control immunosuppression risks. 3. Explore novel blood–brain barrier-opening technologies (e.g., ultrasound-mediated approaches [211,212]) to overcome vector delivery hurdles. 4. Establish reversible editing frameworks (e.g., degron-tagged Cas9 [213,214]) with strict prohibition of germline editing [215,216] to conform with ethical norms. 5. Employ advanced molecular/cellular techniques for mechanistic dissection of regulatory networks—particularly receptor signaling crosstalk and functional signal preference. 6. Develop cross-validated humanized models that accurately simulate human pathophysiology to bridge interspecies gaps and define precise brain-repair therapeutic windows. These systematic endeavors will establish the scientific foundation for implementing the CX3CR1-based triple precision strategy in stroke patients.

### 4.2. Breakthroughs in Cutting-Edge Technologies

Spatial metabolomics technology enables high-resolution dynamic mapping of key metabolites (e.g., lactate) within post-stroke infarct marginal zones [217,218]. Integrating in situ mass spectrometry imaging with metabolic flux analysis, this approach constructs three-dimensional (3D) dynamic atlases of metabolic microenvironments essential for deciphering functional coupling mechanisms. These atlases reveal how lactate fluctuations regulate local HIF-1α signaling and CX3CR1 activity [219], thereby driving the temporal transition of microglia from pro-inflammatory to reparative phenotypes. Consequently, spatial metabolomics holds significant promise for elucidating the spatiotemporal logic governing metabolic reprogramming and neuroimmune signal crosstalk, thereby identifying novel intervention targets for future CX3CR1 dynamic function regulation through energy metabolic pathway modulation.

Breakthroughs in deep learning algorithms—particularly graph neural networks and Transformer architectures—provide powerful computational tools with nonlinear modeling capabilities for integrating massive heterogeneous multi-omics data (including single-cell transcriptomics, spatial proteomics, metabolomics, and multimodal imaging) [220,221]. Dynamic predictive models constructed from such data can learn patient-specific temporal data streams to accurately predict individualized post-stroke CX3CR1 signaling trajectories and identify optimal intervention time windows. This has catalyzed the innovative “artificial (AI)-enabled neuroimmune clock” framework, whose core lies in identifying critical dynamic signatures of neuroimmunological interactions, such as key transition nodes in microglial state transitions [222,223]. This framework establishes both theoretical foundations and precise temporal coordinates for personalized neural repair strategies. Deep learning is driving a fundamental paradigm shift from static omics mapping to dynamic mechanism modeling, ultimately enabling high-dimensional spatiotemporal navigation of neuroimmunoregulation.

In summary, technological innovations now achieve breakthroughs through dual-path empowerment integrating spatial dimensions (metabolic microenvironment profiling) and temporal dimensions (dynamic modeling/prediction). By synergizing these technologies—where spatial metabolomic maps provide high-precision physiological substrates for AI-enabled neuroimmune clocks, while critical kinetic features derived from clock analyses feedback into spatiotemporal optimization of metabolic targets—we will establish a closed-loop system spanning metabolic remodeling to precise cellular state navigation. This will orchestrate a paradigm transition from “stage-dependent” to “individualized spatiotemporal programming” for stroke neural repair.

The above translation challenges and technical breakthroughs can be summarized in Table 3:

## 5. Conclusions

This review delineates the core pathophysiological signature of stroke-induced CX3CR1/CX3CL1 signaling: its spatiotemporally evolving dual neurotoxic/neuroreparative effects. The stage-specific spatiotemporal reprogramming of signaling sources and cellular targets critically steers pathological progression and neurological outcomes. Consequently, effective therapeutic interventions must implement spatiotemporally precise modulation to suppress detrimental pathways while amplifying reparative functions, thereby optimizing neurological recovery and preventing chronic neurodegeneration. This review accordingly proposes a “Triple Precision Strategy” clinical translation framework integrating stroke phase stratification, biomarker profiling, and targeted interventions. However, CX3CR1-targeted therapy’s clinical translation faces persistent bottlenecks: inadequate validation of biomarkers, incomplete elucidation of upstream regulatory mechanisms, and clinically significant interspecies differences. Technological innovations are now breaking through these barriers: spatial profiling technologies decode neuroimmune spatiotemporal logic, while AI-driven dynamic modeling predicts critical intervention points for personalized therapy. Consequently, the deep integration of these technological directions will establish a closed-loop regulatory system, driving the convergence of intervention timing, biomarker-guided staging, and advanced delivery/gene regulation technologies in CX3CR1-targeted therapy. This synergy will ultimately establish a paradigm shift—from stage-dependent protocols toward individualized spatiotemporal precision programming in stroke treatment. In summary, the CX3CR1/CX3CL1 signaling axis serves as the central regulatory mechanism for post-stroke brain repair. Precision modulation of this axis holds transformative potential as a fundamental therapeutic strategy for stroke recovery. Consequently, sustained technological refinement targeting this pathway represents a critical research imperative moving forward.

As this review adopted a narrative review methodology without strict adherence to PRISMA (Preferred Reporting Items for Systematic Reviews and Meta-Analyses) protocols, it may not exhaustively encompass all of the relevant literature. Consequently, readers should interpret the conclusions with critical discernment. Nonetheless, through synthesizing cross-disciplinary evidence and contentious viewpoints, this work constructs an innovative framework for CX3CR1-targeted stroke therapeutics. Our analysis uncovers pivotal knowledge gaps and paradoxical findings, establishing a conceptual foundation for future mechanistic investigations and clinical translation of CX3CR1 pathway modulation.

## Figures and Tables

**Table 1:** Phase-specific dynamics and functional duality of the CX3CR1/CX3CL1 axis in post-stroke pathology.

Phase	CX3CL1 Source	CX3CR1 Dynamics	Beneficial Functions	Detrimental Functions
Hyperacute	Damaged core neurons [7,28]	Rapid upregulation in peri-infarct microglia [5,8]	Mediates microglial migration [33,34] and debris clearance [5,8]	Exacerbates oxidative stress and neuroinflammation [41,42]
Acute	Vascular endothelium [43,44,45]	Overexpression in Ly6C^+^ monocytes [52,53]/microglia [49,50,51]	Induces IL-10^+^ anti-inflammatory polarization [49,50,51]	Drives monocytes infiltration [52,53] → cytokine storm [47,48]
Subacute	Peri-infarct astrocytes [5,71]	Sustained overexpression in microglia/MDMs [6,66]	Phagocytic clearance [6,66], angiogenesis [77], neurogenesis [64,65,81]	Insufficient signaling → glial scarring [67,68,69,70]
Chronic	Neurons and astrocytes [24,83,84]	Stable overexpression in microglia [85,86]	Early phase: Continues repair [4]	Long-term: Pathological synaptic pruning [88,89,90], chronic neuroinflammation [51,91,92], Aβ acceleration [93,94]

**Table 2:** “Stroke phase-biomarkers-targeted interventions” triple precision strategy.

Phase	Biomarkers	Targeted Interventions
Hyperacute	Serum: sCX3CL1↑ + MMP-9↑ [7,118,119]	1. Avoid CX3CR1-targeted therapy [100,101,102,103,104,105]. 2. Core therapy: Intravenous thrombolysis (≤4.5 h)/thrombectomy (≤6 h) [161,162,163]. 3. Adjunct: Short-term non-CX3CR1 immunomodulation [164,165,166].
Acute	CSF: sTREM2↓ + CD36↓ [127,128]	pH-responsive nanocarriers delivering rCX3CL1 to penumbra [169,170,171,172,173,174].
Subacute	PET-MRI: [^11^C]PBR28↑ + microglial P2RY12↑ [131,132]	Intravenous FKN-Fc fusion protein + MSCs [175,176,177,178,179].
Chronic	CSF: sTREM2↑ + NfL↑ [139,140,141,142]	1. AAV-HEXB promoter-driven CRISPR-Cas9 CX3CR1 knockout [186,187,188]. 2. Tamoxifen-inducible regulation [188].

**Table 3:** Clinical translation bottlenecks and tech breakthroughs.

Key Challenges/Breakthrough Areas	Critical Issues/Technical Innovations	Future Directions
Biomarkers Translation Barriers	1. Clinically validated standard definitions and testing thresholds remain unavailable 2. Unresolved core regulatory and upstream mechanisms governing the CX3CR1 signaling axis [8]	1. Large-scale clinical studies to validate biomarker utility 2. Mechanistic dissection of CX3CR1 regulatory networks (transcriptional control, signaling preferences)
Immunosuppression Risk	Imprecise systemic cytokine modulation compromises host defense [194,195,196,197]	Integrate dynamic CD4^+^/CD8^+^ ratio monitoring [210]
Delivery Hurdles	Fibrotic scarring impairs penetration [198,199]	Develop novel BBB-opening tech (e.g., ultrasound-mediated) [211,212]
Gene Editing Ethics	Ethical implications of irreversible editing	Implement reversible systems (e.g., degron-tagged Cas9) [213,214] + Ban germline editing [215,216]
Cross-Species Temporal Scale Discrepancies	Rodent neural repair windows mismatch human disease progression complexity [205]	Humanized model systems (e.g., brain organoids integrated with in vivo imaging) development for precise human brain repair window definition [209]
Spatial Metabolomics Implementation	In situ mass spectrometry imaging with metabolic flux analysis enables 3D microenvironmental mapping [217,218]	Spatiotemporal logic decoding of lactate/HIF-1α/CX3CR1 signaling axis driving microglial phenotypic conversion [219]
Deep Learning-Driven Multi-Omics Integration	Heterogeneous data synthesis via graph neural networks/transformers establishes “neuroimmune clock” frameworks [220,221]	AI-powered prediction of individualized CX3CR1 activity trajectories and optimal intervention windows, defining critical kinetic nodes [222,223]

## Data Availability

No new data were created or analyzed in this study. Data sharing is not applicable to this article.
